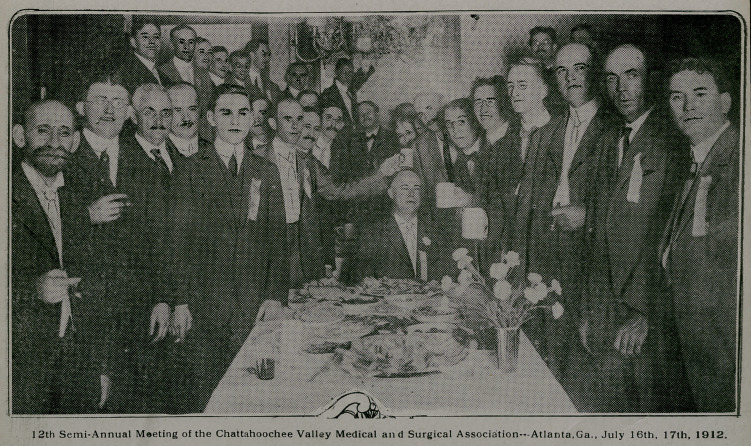# Pin-Point Os Uteri

**Published:** 1912-07

**Authors:** Jno. Prather

**Affiliations:** Ft. Davis, Ala.


### PIN POINT OS UTERI.

By Dr. Jno. Prather, Ft. Davis, Ala.

This condition, I have heard, though I have been unable to see his description, was first described by Dr. Marion Sims, who at one time lived near my present place of residence, then in Montgomery, and then in New York.

The various text-books on Gynecology that I have had access to merely refer to the anomaly or devote only a few lines to its discussion.

One of the prominent surgeons in Montgomery has recently tried an operation for this trouble as well as anteflexions described in his book on Gynecology by Dr. C. E. Dudley, of Chicago.

The term "Pin-point Os Uteri" to me seems a misnomer, as it conveys to the mind an idea of a very small os in a probably normal or stenosed cervix—something closely related to atresia, or the cervical canal. "Pencil-Point Cervix Uteri" would be a better term, as some of these have already been dilated by other doctors in an attempt to relieve the dysmenorrhea present in the cases with stenosis.

Another thing that is not made clear is that a cervix of this shape is always accompanied by more or less anteflexion of the body of the uterus, and whether there is also retroversion or not, the pencil-shaped cervix projects into the vagina in such a manner as to give the impression of prolapse. I have never seen any real prolapse in these cases, though I did diagnose my first ones as prolapse.

In no case of anteflexion in nullipara or virgin have I ever seen the patient complain of irritable bladder except after dilation or curettage. Sometimes on close questioning they will give a history of having had such trouble for a week or two, probably for some other cause.

The persistence of the infantile anteflexion, the smallness of the cervix, the presence and history of hookworms, the intermittent malaria, and the extreme anaemia, all present in a sterile adult, seems to show some relation between these infections and the malformation. Then we know that boys do not reach the state of puberty until late in their teens when infected with hookworms and that girls no not menstruate very often when anaemic.

Outside of the dysmenorrhea present in most of these cases and the sterility in the extreme cases, the symptoms are usually those of some other trouble or troubles.

In this particular locality you can find women in quantity with varying degrees of this malformation. The mild ones bear one child and quit. The extreme ones never conceive. They are extremely apt to miscarry. They begin to have leucorrhea in early girlhood. The hookworms are easy to get out and they do come out in large quantities, but the malaria persists in spite of large doses of ordinary quinine. The attacks return every few months after you had thought the parasites were dead. I have found only one successful way to eliminate malaria in these cases. Six grains of quinine sulphate dissolved in six drops of pure hydrochloric acid, then dilute with water and take before meale. Their ears will roar for th first time in their lives.

Nearly all these cases have chronic colitis, especially those with extreme anteflexion with the cervix pressing at right angles on the rectum, and those with a slight anteflexion and extreme retroversion. They are easily relieved of this complication, but just as easily relapse.

The improvement in health, flesh and spirits after relieving the anaemia is wonderful and some of the mild malformations will not remain sterile after the general health is improved.

Anything I have to do in a surgical way to the uterus is postponed until the general health is improved, except the menorrhagia, which is sometimes very prolonged.

I submit ten cases which I have been treating off and on for two or three years. Their general health has been improved but they are still under occasional treatment with one or two exceptions.

Case 1.—This is my first case, seen three years ago near Seale, Ala. I have lost track of her lately.

Age 25, anaemic, married four years, fairly well-to-do negro family, childless; slight anteflexion with retroversion and acute endometritis. This abdominal walls were thin with a prominently murmuring abdominal aorta. Prominent symptoms cleared up after hookworm treatment and hygienic vaginal measures. The cervix was long, pointed and hard.

Case 2.—Age 22, married two years, childless, complaining of pain over pulsating, murmuring right carotids. Haemoglobin 45%. First two weeks after hookworm treatment, 65%. Next two weeks, 75%. Kept pain relieved with iodids and mild vegetable arterial sedatives. No special complaint of malformation which was an average one of this type. She now has syphilis.

Case 3.—This was a young colored woman, age 26, married six years. First child was choked by cord during labor. Then she miscarried twins at eight months. About a year after that she came to me with symptoms characteristic of extreme anteflexion, hard-pointed os, colitis, malaria, and hookworms, and if others had not corroborated her history of having borne children I would not have believed her, as her uterus was so different from other anteflexions I had seen following childbirth. After hookworm and dilatation of cervix and occasional treatment for intermittent malaria, she fattened up and looked well as she does now, but only the other day after two years I had to give her quinine in acid solution and recommend normal salt enemas for the persisting colitis.

Case 4.—Stout, healthy looking negro woman. Husband wants her to have children. His second wife, first one prolific.

Married ten years. Uterus, a typical one of this class, pointed cervix and nothing extreme in way of anteflexion or retroversion. No symptoms. Offered to dilate cervix but did not insist on it and no treatment at all was ever attempted.

Case 5.—This is a young colored woman whom I treated in 1909 for endometritis by hot douches and for hookworms. Aparent recovery. In 1910 she had symptoms of severe appendicitis and at that time the cervix was small, pliant and healthy, no discharge or pelvic adhesions present. She relapsed on return to solid food, but soon returned to excellent health, except for intermittent malaria, which is characteristic of the whole household. Her young sister is having quite a good deal of trouble with her menses. And her mother's sister lost her mind at the menopause, lingered a couple of years and died childless. In 1912 she has had a relapse from the appendiceal trouble. she is childless, but wants children.

Case 6.—This case is of interest in that there were some facial features of the other cases resembling childhood or lack of development; this young colored woman has the hard features of those suffering from lack of sufficient thyroid secretion. She has suffered with lecorrhea and dysmenorrhea since early girlhood. Was thin, ashy looking and anaemic, had hookworms in quantity and intermittent malaria. There is extreme anteflexion and the hard-pointed cervix presses on the rectum, causing colitis and constipation; no retroversion. Great improvement from hookworm and malarial treatment but not much from dilation. Colitis constantly relapsing. Still has leucorrhea after two years from beginning treatment. Of fairly chaste habits.

Case 7.—Age 26, married five years. Leucorrhea and dysmenorrhea since early girlhood. Intermittent malaria. Will not take hookworm treatment. About three years ago gave birth to eight month's still-born child. Lacerated cervix slightly and slightly injured fibrous and muscular coat of upper half of vagina. Was curetted by one doctor and operated upon soon after by another for suppurating appendix. Has endometritis with each menstrual period. Anteflexion, retroversion and leucorrhea, and long-pointed cervix with large canal. Is one of several other cases I will not enumerate who have borne one child, or have had several miscarriages, and have now decided to let doctors alone and be content without children. I have never treated this patient except in her acute malaria or endometritis. When she gets better she quits taking medicine, so in a few months has another chill.

Case 8.—Young married colored woman. Had been married two or three years. Good health in early girlhood. Has sterile aunt, on mother's side. Was anaemic, painful, liver swollen very greatly, feverish, menstruating three weeks and missing one. Confined to bed for three months. Hookworm treatment, malarial treatment, fractional doses of silver nitrate, etc., got her up in about a month and she now, two years after, enjoys excellent health, but is still sterile.

There was both anteflexion and retroversion, the cervix hard and pointed and the uterus badly swollen.

Other Cases.—I have on hand a number of young colored girls with difficult menstruation, most of whom improve after elimination of hookworms and malaria. I do not make digital examinations in these cases, but rely on suprapubic examination to be fairly sure they are not pregnant. It will take several years to tell whether any of these are sterile or not. When they still have trouble after the elimination of hookworms and malaria, I recommend daily hot normal salt injections but only a few will take them. One of these patients had vicarious nasal menstruation and the ignorant negro parents became angry because I made her take hookworm treatment. They also refused to use the douches which I ordered in the hope that they would stimulate the growth of the uterus. After thirteen months I hear that she looks a lot better but is still bleeding at the nose. However, most of these girls of the colored race who still have trouble after the blood is thickened are of chaste habits and probably will continue to have some trouble for a few years yet, but I hope not as much trouble as they would have had if the anaemia had not been remedied.

## Figures and Tables

**Figure f1:**